# Inflammatory Mediators and Insulin Resistance in Obesity: Role of Nuclear Receptor Signaling in Macrophages

**DOI:** 10.1155/2010/219583

**Published:** 2010-05-20

**Authors:** Lucía Fuentes, Tamás Rőszer, Mercedes Ricote

**Affiliations:** Department of Regenerative Cardiology, Centro Nacional de Investigaciones Cardiovasculares, Instituto de Salud Carlos III, C/Melchor Fernández Almagro 3, 28029 Madrid, Spain

## Abstract

Visceral obesity is coupled to a general low-grade chronic inflammatory state characterized by macrophage activation and inflammatory cytokine production, leading to insulin resistance (IR). The balance between proinflammatory M1 and antiinflammatory M2 macrophage phenotypes within visceral adipose tissue appears to be crucially involved in the development of obesity-associated IR and consequent metabolic abnormalities. The ligand-dependent transcription factors peroxisome proliferator activated receptors (PPARs) have recently been implicated in the determination of the M1/M2 phenotype. Liver X receptors (LXRs), which form another subgroup of the nuclear receptor superfamily, are also important regulators of proinflammatory cytokine production in macrophages. Disregulation of macrophage-mediated inflammation by PPARs and LXRs therefore underlies the development of IR. This review summarizes the role of PPAR and LXR signaling in macrophages and current knowledge about the impact of these actions in the manifestation of IR and obesity comorbidities such as liver steatosis and diabetic osteopenia.

## 1. Introduction

Progressive development of insulin resistance (IR) is a prediabetic state which is today a widespread metabolic abnormality of adults and adolescents in industrialised societies [1]. Impaired insulin action is considered the first stage of type 2 diabetes mellitus (T2DM). The consequences of IR manifest at many levels and in many metabolic processes, producing a cluster of homeostatic abnormalities including glucose intolerance, overt hyperglycemia, hyperinsulinemia, and atherogenic dyslipidemia, collectively referred to as metabolic syndrome (MetS). Liver steatosis, kidney disease, and osteoporosis are also frequent comorbidities of T2DM and MetS [2–4]. 

**Figure 1:**
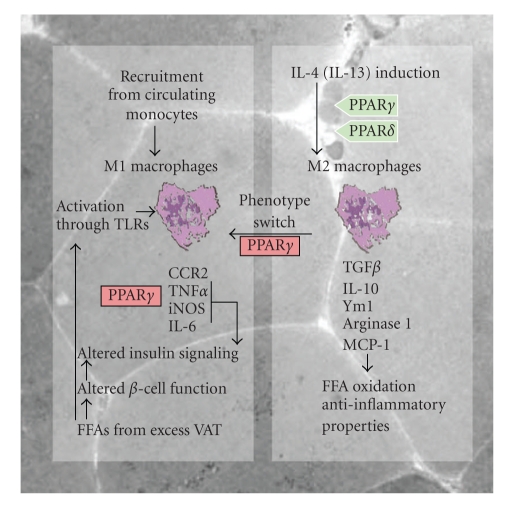
Cytokine release by adipose tissue macrophages contributes to insulin resistance. Free fatty acids (FFAs) released from visceral adipose tissue (VAT) promote polarization toward the M1 phenotype through activation of Toll-like receptors (TLRs), and also impair insulin secretion and action. Differentiation to the M1 phenotype is inhibited by PPAR*γ* signaling, and activation of PPAR*γ* or PPAR*δ*, in response to IL-4, promotes polarization toward the M2 phenotype. IL-13 is also suggested to be involved in the M2 phenotype switch. Inflammatory monocytes migrating into VAT can also differentiate into M1 macrophages. Inflammatory mediators produced by M1 ATMs alter insulin responsiveness. CCR2: chemokine receptor-2, TNF*α*: tumor necrosis factor, iNOS: inducible nitric oxide synthase, IL-6: interleukin-6, TGF-*β*: transforming growth factor beta, IL-10: interleukin-10, Ym1: secretory chitinase protein-1, MCP-1: monocyte chemoattractant protein-1 (MCP-1).

IR correlates positively with obesity, and the rapidly growing incidence of T2DM and MetS is therefore often attributed to lifestyle factors such as excess caloric intake and insufficient physical exercise in urbanized human populations [5]. The main predisposing factor for IR is intra-abdominal accumulation of adipose tissue (AT), which leads to central obesity [5, 6]. The total load of visceral adipose tissue (VAT) and the rate of free fatty acid (FFA) mobilization from VAT to the portal venous system are well-established correlates of IR and high circulating levels of insulin [7–9]. Several mechanisms link visceral adiposity and elevated FFA levels to IR. The elevated VAT mass liberates excess amount FFAs to the bloodstream, which contribute to muscle and liver IR by triggering reduced insulin signaling and increased hepatic gluconeogenesis. High levels of FFA shift the substrate preference of mitochondrial oxidation from glucose to FFA, and this can diminish the insulin secretory response to glucose of islet *β*-cells, leading to relative insulin insufficiency [10, 11]. Moreover, FFAs induce an inflammatory response in macrophages, adipocytes, and muscle cells via toll-like receptor (TLR) activated pathways (Figure 1). Modified lipoproteins such as oxidized and glycated low-density lipoproteins derived from excess VAT can accumulate in certain tissues, including subendothelial spaces, muscle cells, liver or kidney mesangium and tubular epithelial cells, where they can give rise to atherogenesis, lipotoxic injury, and inflammation [12]. VAT is also an active endocrine organ able to secrete a wide variety of inflammatory cytokines with key functions in the development of IR [13].

In recent years, macrophages have been recognized as major sources of proinflammatory mediators, which are largely responsible for the manifestation of IR. Macrophages are plastic cells and their ability to produce cytokines is determined by their phenotype. The so-called classical activated or “M1” macrophages secrete high amounts of inflammatory mediators while the alternatively activated “M2” macrophages are low cytokine producers. In obesity the balance between M1 and M2 macrophages is disturbed. Thus, production of inflammatory cytokines by VAT macrophages increases significantly [14]. This situation creates a general subclinical inflammatory state [15] that will ultimately lead to altered insulin responsiveness. Recent studies reveal that macrophage activation is regulated by lipid metabolites through the activation of nuclear receptor transcription factors, and that imbalances in macrophage nuclear receptor signaling can lead to IR [13]. 

Nuclear receptors (NRs) are a superfamily of ligand-activated transcription factors that control transcription of their target genes through direct or indirect mechanisms. Directly, NRs bind to specific DNA sequences in *cis*-regulatory elements within promoter regions, activating or repressing target gene expression by recruiting or releasing coactivators and corepressors [16]. Indirectly, NRs can *trans*-repress the transcription of certain genes controlled by other transcription factors, such as nuclear factor kappa-B (NF-*κ*B) or activator protein-1 (AP-1) [17, 18]. Prominent members of the NR superfamily are peroxisome proliferator activated receptors (PPARs), activated by FFAs, eicosanoids, and prostaglandins, and liver X receptors (LXRs), activated by cholesterol metabolites. These “lipid sensors” appear to play a central role in the control of lipid metabolism. NRs are moreover the targets of environmental obesogens such as phtalates, organotins, bisphenol A, and xenobiotics that interfere with NR signaling and which are thought to underlie the spread of obesity and its comorbidities [19]. In addition, evidence acquired over the last decade demonstrates that PPARs and LXRs have important antiinflammatory effects and can control macrophage activation, suggesting potential in the medication of IR.

The role of NRs in linking metabolism and inflammation is especially relevant to the pathogenesis of obesity-induced IR. Synthetic pharmacological ligands for PPAR*γ* (thiazolidinediones; TZDs) and PPAR*α* (fibrates) are used clinically due to their hypolipidemic and insulin-sensitizing properties. Additionally, pharmacological activation of LXRs results in increased HDL levels and net cholesterol loss, therefore, synthetic LXR ligands have a potential medical benefit to treat dyslipidaemias and atherosclerosis. A growing body of literature suggests that these drugs, due to their antiinflammatory effects, can have a broader impact in metabolic diseases, especially in obesity comorbidities. Here we summarize the latest findings linking IR, inflammatory mediators, and macrophages and discuss the regulatory role of NR signaling in macrophage cytokine production associated with obesity and obesity comorbidities.

## 2. Friend or Foe? M1 and M2 Macrophages in Adipose Tissue

Over the last few years, understanding of macrophages as an important element of IR development has advanced considerably with the identification of distinct functional macrophage subsets. Macrophages have a highly plastic phenotype that allows them to specialize and display polarized functional properties, such as inflammatory or antiinflammatory actions in response to cytokines and microbial products. Macrophage polarity can be determined by T-helper cells. Cytokines released by T-helper 1 (Th1) cells, such as interleukin-2 (IL-2), gamma-interferon (IFN*γ*), and tumor-necrosis factor alpha (TNF*α*), induce the classical macrophage phenotype, activating them to stimulate cellular immunity and inflammation. Th1 cells also secrete granulocyte-macrophage colony stimulating factor (GM-CSF), which promotes medullar monocyte/macrophage differentiation. In contrast, T-helper 2 (Th2) cells secrete interleukin-4 (IL-4) and interleukin-13 (IL-13), which induce an alternative phenotype by attenuating macrophage-mediated secretion of inflammatory mediators and instead inducing macrophage programs for FFA oxidation [20]. Adapting the Th1/Th2 nomenclature, Mantovani and colleagues in 2002 started to refer to polarized macrophages as M1 and M2 cells [21]. M1 macrophages are activated proinflammatory cells, while M2 macrophages are characterized by an antiinflammatory phenotype. Although there is a clear association of obesity and IR with macrophage infiltration of AT and M1 macrophage activation, the dominant phenotype of adipose tissue macrophages (ATMs) is still an open debate.

The first evidence suggesting diversity of ATM phenotype was obtained from chemokine receptor-2 (CCR2) knockout (KO) mice (CCR2KO) [22]. CCR2 is a cell-surface receptor for monocyte chemoattractant protein-1 (MCP-1), a chemokine which specifically mediates monocyte chemotaxis. Under normal physiological conditions, the ATM content of CCR2KO AT does not differ from wild-type AT, and CCR2KO mice show no overt metabolic alteration. However, CCR2KO mice fed a high-fat diet accumulate fewer ATMs in AT than similarly fed wild-type mice, and present an attenuated inflammatory profile and greater insulin sensitivity. Thus metabolic challenge with supernormal fat intake triggers macrophage recruitment to VAT via a MCP-1/CCR2-dependent process, but CCR2 is not required for resident macrophage recruitment. Brake and co-workers subsequently identified CD11c-positive (CD11c^+^) and CD11c-negative (CD11c^−^) macrophage populations in mouse AT [23]. The numbers of CD11c^+^ cells increase in response to a high-fat diet, and this is accompanied by increased AT expression of transcripts for CCR2, interleukin-6 (IL-6), and intercellular adhesion molecule I (ICAM-I), a leukocyte adhesion receptor needed for macrophage tissue infiltration. CD11c^+^ cells were thus proposed as an inflammatory macrophage population in AT. Interestingly, conditional bone marrow depletion of CD11c^+^ cells in obese mice results in a rapid normalization of insulin sensitivity [24]. Moreover, further studies confirmed that ATMs recruited to AT in diet-induced obesity express high levels of IL-6, inducible nitric oxide (NO) synthase (iNOS) and CCR2, all characteristics of the M1 phenotype [25, 26]. In obese mice chronic iNOS blockade attenuates high-fat diet induced IR and, similar to CCR2KO mice, reduces macrophage VAT infiltration, as shown by lowered mRNA expression of MCP-1 and the macrophage cell surface receptor CD68 [27]. In addition, resident ATMs show very low (if any) inflammatory activity and express many M2-associated genes such as arginase 1, interleukin-10 (IL-10) and the secretory chitinase protein Ym1 [28]. 

The identification of two monocyte populations in mouse blood [29, 30] supported the hypothesis that M2 macrophages resident in AT are descendents of circulating nonactivated monocytes, while M1 macrophages derive from a population of circulating inflammatory monocytes that are recruited to AT where they continue their differentiation and orchestrate the inflammatory response. This model is further supported by the observation that, blood mononuclear cells from obese patients are in a proinflammatory state [31].

An alternative hypothesis is that M1 macrophage polarization during obesity progression occurs via in situ reprogramming of ATMs from an M2 to an M1 phenotype. In vitro, it is well established that the pattern of macrophage function depends on the agonist to which they are exposed [32]. For instance, in vivo, treatment of tumor-bearing animals with interleukin-12 (IL-12) shifts tumor-associated macrophages from a dominant M2 profile (elevated expression of TGF*β*, IL-10, and MCP1) to a proimmunogenic/inflammatory M1 profile (elevated expression of IL-6 and TNF*α*) [33]. However, it remains uncertain whether this *“*in situ*”* phenotype switching can also occur in AT (Figure 1). 

Interestingly, PPAR*γ* and PPAR*δ* have been recently implicated in the transcriptional regulation of monocyte/macrophage phenotypic shift (Figure 1). Using myeloid-specific PPAR*γ* and PPAR*δ* KO mice (Mac-PPAR*γ*KO and Mac-PPAR*δ*KO), Odegaard et al. showed that PPAR*γ* and PPAR*δ* are both necessary for optimal induction of the M2 macrophage phenotype by IL-4 (a classical Th2 cytokine) [34, 35]. However, these factors make distinct contributions to this process: PPAR*γ* is specifically required for IL-4-dependent activation of fatty acid oxidation, whereas PPAR*δ* is required for the full expression of the IL-4-dependent immune phenotype (Figure 1). Furthermore, the AT of fat-fed Mac-PPAR*γ*KO accumulates fewer macrophages and shows lower M2-related gene expression than the AT of fat-fed wild-type mice. However, fat-fed Mac-PPAR*γ*KO mice are more obese, indicating that the reduced number of M2 macrophages leads to major alterations in adipocyte metabolism [34]. These studies demonstrate that activation of PPAR*γ* and PPAR*δ* in ATMs ameliorates IR not only through the regulation of cytokine production but also by modulating ATM phenotype.

## 3. Nuclear Receptor Signaling Reduces Cytokine Production by ATMs and Ameliorates Insulin Resistance

The paracrine and endocrine functions of VAT actively contribute to the development of IR. VAT is a major source of a wide variety of cytokines produced mainly by macrophages and of certain hormone-like factors produced by adipocytes. The best known VAT-produced cytokines include C-reactive protein (CRP), IL-6, interleukin-1 (IL-1), interleukin-18 (IL-18), and tumor necrosis factor (TNF*α*) [36]. These inflammatory mediators exert their actions not only on AT cells, but also on other cell types such as hepatocytes, liver Kuppfer cells, kidney mesangial cells, osteoclasts, and muscle fibers. Indeed, in T2DM patients, elevated VAT expression of TNF*α* is associated with the onset of IR, and high circulating levels of interleukin-1 receptor antagonist (IL-1ra) and TNF*α* correlate strongly with MetS in human populations [37]. The mechanisms by which inflammatory cytokines produce defects in insulin signaling are not fully understood; however, many studies suggest an origin in insulin postreceptor signaling. Binding of insulin to its receptor is followed by phosphorylation of the insulin receptor substrates IRS-1 and IRS-2. Tyrosine phosphorylation of IRS-1 and IRS-2 mediates insulin signaling; however, serine phosphorylation of IRSs can block downstream signaling. There are thus two pathways by which cytokines appear to interfere in insulin signaling: by impairing IRS tyrosine phosphorylation or by inducing IRS serine phosphorylation [38]. For instance, TNF*α* impairs tyrosine phosphorylation mediated by PI3-kinase, leading to insufficient glucose uptake by muscle cells [39, 40]. In addition, there is evidence implicating the serine kinases c-Jun Kinase (JNK) and inhibitor of NF-*κ*B kinase (IKK) in cytokine-dependent IR: obesity is associated with increased JNK activity in adipose and liver tissues [41] and mice lacking IKK-*β* are resistant to obesity-induced IR [42]. These kinases also affect AP-1 and NF-*κ*B transcription factors, promoting further inflammatory gene expression. In addition, SOCS proteins, another class of inflammatory mediators, have been found to be involved in obesity-induced IR. SOCS proteins block insulin signaling either by interfering with IRS-1 and IRS-2 phosphorylation or by targeting IRSs for proteosomal degradation [43, 44]. 

Studies on PPARs and LXRs indicate that these nuclear receptors are important regulators of proinflammatory cytokine production by macrophages. In LPS- or IFN*γ*-stimulated macrophages, activation of PPAR*γ* represses the induction of inflammatory genes including iNOS, IL-6, cyclooxygenase-*2 (*COX-2), and matrix metalloproteinase 9 (MMP9) [45, 46]. Activation of LXRs represses almost the same genes as PPAR*γ* [47, 48], while PPAR*α* shows a distinct pharmacological profile, inhibiting expression of tissue factor [49]. Finally, PPAR*δ* deficiency in macrophages is associated with low levels of MMP9 and MCP1 [50]. Recently, increased cytokine production has been reported in vivo in the AT, liver, and muscle of myeloid PPAR*γ*KO mice, correlating with the development of IR in these animals [51]. 

Most evidence indicates that the basic mechanism underlying the antiinflammatory actions of NRs is interference in AP-1/NF*κ*B signalling [17, 18]. However, alternative pathways are not excluded. For example, mechanisms involving blockade of the clearance of corepressor complexes from promoters have recently been proposed. These processes are thought to involve SUMOylation of PPAR*γ* or LXRs [52]. There thus appears to be no single mechanism of repression, and pathway selection seems to depend on the signal, the NR isoform involved, and even the gene promoter. 

The ability of PPAR and LXR receptors to control macrophage-mediated inflammation by these mechanisms appears to have an important impact on the control of IR. Indeed, the beneficial effect of weight loss on obesity-related IR might be associated with an improved inflammatory profile in the stromal vascular fraction of AT, which contains the ATMs [53]. 

Unlike other AT related proteins, the adipocyte protein, adiponectin, contributes to the maintenance of insulin sensitivity and seems to be able to antagonize the proinflammatory effects of macrophages [54, 55]. Adiponectin is the most abundant adipocyte-derived factor in the circulation and low levels of this protein are linked to high body mass index, IR, dyslipidemia, and increased risk of cardiovascular disease [56]. Consistently, adiponectin immunostaining is reduced in the AT of mice fed a fat- and carbohydrate-rich diet [57]. In humans, a marked gender difference in AT distribution evolves during puberty, resulting in elevated VAT mass and lower adiponectin production in adult males and an associated higher susceptibility to insulin signaling defects [58, 59]. Moreover, TNF*α* reduces adiponectin production [60]. Importantly, adiponectin also has a potent antiinflammatory action on macrophages, suppressing lipopolysaccharide (LPS)-stimulated cytokine production possibly via the antiinflammatory IL-10 signaling pathway [61]. Adiponectin promoter is regulated by PPAR*γ*/RXR*α* heterodimers, and administration of TZDs has been reported to significantly increase plasma adiponectin concentrations in insulin-resistant humans and rodents without affecting their body weight [62]. Activation of PPAR*γ* induces production of adiponectin not only from adipocytes but also from skeletal muscle, which augments the antidiabetic actions of PPAR*γ* [63, 64].

## 4. Liver Resident Macrophages Link Obesity to Steatosis

The liver is responsible for the coordination of intermediate metabolism. Hepatocytes are actively involved in glucose and lipid metabolism (including cholesterol and lipoprotein synthesis), plasma protein synthesis, and the production of inflammatory proteins such as CRP [65]. Obesity is associated with a high incidence of steatosis, a pathological accumulation of lipids within hepatocytes. Nonalcoholic fatty liver disease (NAFLD) is the most common form of chronic liver disease and is characterized by excess liver lipid accumulation and hepatic IR. At a later stage of disease progression, NAFLD can occur with hepatic inflammation, leading to nonalcoholic steatohepatitis (NASH) and culminating in hepatic fibrosis or cirrhosis [3].

In obesity, inflammatory cytokines (IL-1*β*, TNF*α*, and IL-6) and adiponectin released from AT reach the liver through the portal vein and can directly interfere with liver functions. In an inflammatory state, TNF*α* can trigger hepatocyte apoptosis and the activation of the fibrogenic response in stellate cells [66], while IL-6 is implicated in the induction of the acute phase response by eliciting transcriptional activation of CRP [67]. Adiponectin, unlike cytokines, appears to have a protective effect in the liver. Adiponectin administration ameliorates steatosis, probably via inhibition of TNF*α* signaling. In addition, adiponectin exerts antifibrogenic effects: adiponectin KO mice exposed to CCL4 develop more severe fibrosis than wild-type animals [68]. 

However, the most important sources of inflammatory cytokines within the liver are Kupffer cells, the resident macrophages in the liver. Kupffer cells mostly localize in the liver sinusoids but can also migrate into the space of Disse. Like all macrophages, they show phenotypic plasticity, presenting different morphology and functions depending on their intralobular position. Kupffer cells located in the periportal zone are large cells with high phagocytosis capacity and high lysosomal protease activity, whereas Kupffer cells in mid-zonal and perivenous areas are smaller and have lower protease activity [69, 70]. During steatosis, the recruitment of new macrophages into the liver can alter cell distribution, thereby also changing Kupffer cell morphology and function. Indeed, in the livers of NASH patients, enlarged Kupffer cells occur in aggregates around the perivenous regions, while in simple steatosis their distribution is more diffuse [71]. There is also evidence that Th1 immune response dominates in NAFLD, promoting the classical M1 activation of Kupffer cells [72, 73]. In addition, recent studies in rodents suggest a direct role for Kupffer cell M1 activation in hepatic fatty acid metabolism and steatosis [74, 75]. Interestingly, Kupffer cells from rats, fed a high-fat diet or challenged with endotoxin, produce high levels of NO and the TNF*α* M1 cytokine [76, 77]. Moreover, the depletion of Kupffer cells prevents steatosis and the development of insulin resistance [78]. In mice, Kupffer cell depletion is also associated with a decrease in hepatic triglyceride levels and increased expression of key genes involved in fatty acid oxidation, such as PPAR*α* [79]. The ability of PPARs and LXRs to reduce cytokine production in activated inflammatory monocyte-macrophage cells is well documented [45–47]. However, the effects of nuclear receptor agonists on activated Kupffer cells remain unclear. Some studies show that pioglitazone, a clinically available ligand of PPAR*γ*, prevents endotoxin-induced liver injury via a mechanism dependent on suppression of TNF*α* and NO production by Kupffer cells [76, 77]. In mouse liver, PPAR*α* activation is associated with Kupffer-cell mediated reactive oxygen species production and carcinogenesis [80]. Moreover, case reports indicate that therapeutic use of PPAR*α* ligands can lead to hepatic fibrosis [81]. Contrary to these observations, PPAR*α* upregulation has been shown to ameliorate experimentally-induced liver steatosis in rats [82]. Thus the common thread linking PPAR*α* activation, Kupffer cells, and hepatic pathologies remains undefined [83]. 

**Figure 2:**
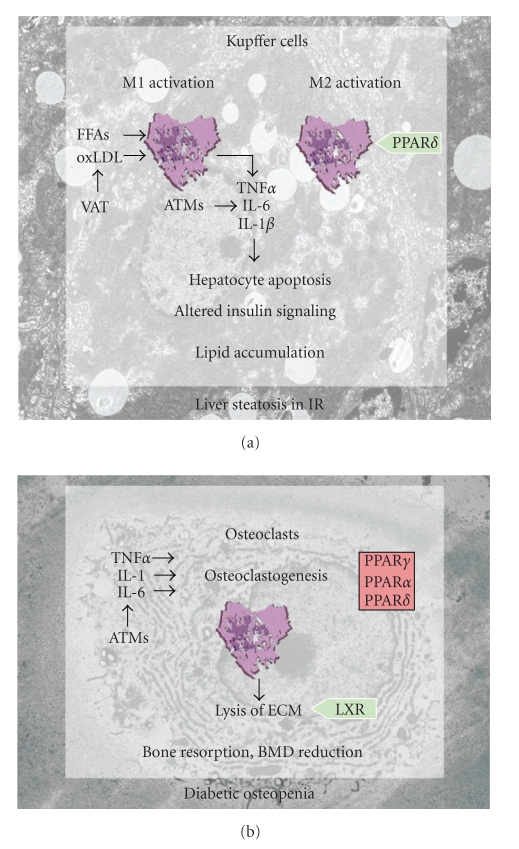
Tissue-resident macrophages are sources and targets of inflammatory mediators in obesity. (a) Liver-resident macrophages (Kupffer cells) are major sources of inflammatory cytokines in obesity and IR. Free fatty acids (FFAs) and oxidized low density lipoproteins (oxLDL) released from VAT promote M1 phenotype polarization through activation of TLRs. The switch to the M2 phenotype is promoted by PPAR*δ* signaling. Inflammatory mediators (IL-6, TNF*α*, and IL-1*β*) originating from M1 Kupffer cells or adipose tissue macrophages (ATMs) induce hepatocyte apoptosis, IR, and lipid accumulation. (b) Osteoclastogenesis is induced by ATM-derived inflammatory cytokines in obesity and IR. Activation of PPARs blocks osteoclastogenesis and impedes bone loss, while LXR promotes osteoclast resorptive activity.

Kupffer cells can also be alternatively activated, and PPAR*δ* has recently been shown to be required for this M2 activation of Kupffer cells (Figure 2(a)). Expression of M2 related genes in Kupffer cells is reduced in PPAR*δ*-deficient lean mice, and transplantation of PPAR*δ*-null bone marrow into wild-type mice is enough to trigger hepatic dysfunction and systemic IR [35], probably due to reduced M2 activation of resident hepatic macrophages. In a similar way, myeloid-specific PPAR*δ* ^−/−^ mice fed a high-fat diet gain more weight, acquire a higher body weight/liver weight ratio, and have a more profound steatosis than control animals [84]. Moreover, M2 markers are downregulated in these animals. PPAR*δ* is thus an interesting potential pharmacological target for the induction of M2 activation to control inflammation and improve steatosis in NAFLD.

## 5. Osteoclastogenesis in Obesity Leads to Bone Mass Reduction

Clinical studies indicate that IR conditions such T2DM and severe obesity are associated with increased fracture risk although not always with low bone mass [2, 85–87]. Despite this association, T2DM has been classically coupled to higher bone mineral density (BMD) [88]. It is likely that in humans diabetic bone is more fragile due to changes in bone architecture rather than as a consequence of the reduced BMD. Furthermore, leptin-deficient obese (ob/ob) mice, a model of obesity and IR, have a complex bone phenotype, displaying increased trabecular bone volume in the spine but short femora with reduced cortical thickness and reduced trabecular volume [89]. Therefore, although IR is clearly associated with bone fragility, a direct effect of IR on BMD is highly controversial. There is also a disputed association of obesity-associated bone fragility with several IR-derived defects, such as high insulin levels, low insulin-like growth factor-1 synthesis, low serum adiponectin, and elevated levels of inflammatory cytokines.

Under IR conditions, a compensatory hyperinsulinemia develops. Insulin appears to be anabolic for bone, and recent clinical studies demonstrate that elevated insulin levels can increase BMD [90]. Adiponectin serum levels decrease with obesity, but osteoblasts and osteoclasts express receptors for adiponectin [91, 92], indicating a direct role of this factor in the regulation of bone homeostasis. Some reports linking obesity with increased BMD have demonstrated that adiponectin can promote bone resorption [93, 94]. In contrast, a recent study reports that adiponectin inhibits osteoclastogenesis in primary human cells in vitro and stimulates osteoblast growth [95]. These contradictory results suggest that the direct action of adiponectin on bone increases BMD, but that the final sum of its direct and indirect actions leads to bone mass reduction. Moreover, bone architecture and mechanical properties unrelated to BMD can be impaired in patients with T2DM, possibly due to the lowered levels of insulin-like growth factor-1, a characteristic alteration in systemic IR. Finally, inflammatory cytokines, including CRP, IL-1, IL-6, and TNF*α*, accelerate bone turnover and osteoclastogenesis, and may lead to reduced BMD in humans [96–99]. Indeed, cytokine plasma levels can predict bone resorption in aged adults [100]. These studies illustrate the complexity of bone physiology and its paracrine/endocrine metabolic control, which makes it difficult to clarify the relationship between low bone mass, obesity, and IR.

Nevertheless, increased osteoclast activity and decreased osteoblast differentiation are the basis of BMD loss. Bone homeostasis is maintained by the equilibrium between the activities of bone-forming osteoblasts and bone-resorbing osteoclasts. Osteoclasts are derived from haematopoietic myeloid bone marrow progenitors whereas osteoblasts and adipocytes originate from bone marrow mesenchymal stem cells [101, 102]. Given the importance of crosstalk between macrophages and adipocytes in obesity progression, the fact that macrophages and osteoclasts, and likewise adipocytes and osteoblasts, share common precursors suggests the existence of important interactions between bone and fat. Osteoclasts and osteoblasts also produce factors capable of influencing AT biology, such as osteocalcin or osteopontin. Osteocalcin is secreted by osteoblasts and modulates the expression of various genes in adipocytes and insulin secreting *β*-cells in pancreatic islets [103]. Osteopontin, which is produced by various cell types such as macrophages, hepatocytes and osteoclasts, promotes inflammation and macrophage accumulation in AT [104]. These findings suggest that bone has endocrine functions through which it might be involved in obesity progression [105]. However, there is little published research into the possible contribution of crosstalk between fat and bone to the regulation of energy balance.

Paradoxically, administration of the insulin sensitizing synthetic ligands of PPAR*γ* can induce bone loss and increase the risk of bone fractures [106, 107]. In mice, activation of PPAR*γ* with TZDs promotes osteoclast differentiation and consequent bone resorption [107]. Consistently, macrophage-specific deletion of PPAR*γ* leads to elevated BMD due to altered osteoclast activity [108]. It is also documented that PPAR*γ* can control osteoblast differentiation from common bone marrow mesenchymal precursors of the osteoblast/adipocyte lineages [109]. Mice with PPAR*γ* haploinsufficiency therefore also have high BMD, coupled with reduced bone marrow adiposity [110]. LXRs are also required for a correct osteoclast function (Figure 2(b)). LXR KO mice show a significantly increased BMD coupled to paradoxically elevated number of osteoclasts in cortical bone, suggesting that LXRs promote osteoclast resorption activity but is not necessary for osteoclast differentiation [111]. Conversely, ligands of PPAR*γ*, PPAR*α*, and PPAR*δ* were recently shown to inhibit the formation of multinucleated osteoclasts from human blood monocytes in vitro [112] (Figure 2(b)).

The use of insulin sensitizing and hypolipidemic drugs, such as PPAR and LXR ligands, might, by decreasing BMD, be related to the increased fracture risk observed in T2DM patients. However, it is still unknown to what extent BMD contributes to increased fracture risk and whether IR has a direct effect on BMD.

## 6. Concluding Remarks

Insulin resistance is the fundamental cause of a broad range of metabolic abnormalities including glucose intolerance, overt hyperglycemia, hyperinsulinemia, atherogenic dyslipidemia, cardiovascular diseases, kidney disease, liver steatosis, and osteoporosis. Obesity-associated chronic inflammation is a key contributor to decreased insulin signaling throughout the disease progression, although the specific mechanisms that link inflammation to IR remain not fully understood. 

The latest advances in the understanding of macrophage biology place macrophages as the drivers of this inflammatory response. Recruitment of M1 inflammatory macrophages and increased cytokine production in AT and liver not only perpetuate inflammation in these organs but also influence other tissue functions. For instance, obesity-associated inflammatory effects on bone physiology are well documented in many clinical studies. However, the results are controversial and difficult to interpret, and there is therefore a need for further studies to address this question and clarify whether IR has direct effects on bone homeostasis. 

The functions of PPAR and LXR nuclear receptors in macrophages include the control of both metabolic and inflammatory pathways. Activation of these receptors thus acts as a link between these two processes closely related to the development of IR. A large body of evidence clearly shows that the insulin-sensitizing properties of NRs are, at least in part, a result of inflammatory control in macrophages. A better understanding of the molecular mechanisms by which NRs control macrophage activation would therefore facilitate the development of pharmacological strategies to specifically target pathways regulating obesity before the onset of obesity-associated complications.
